# Fluorene‐containing tetraphenylethylene molecules as lasing materials

**DOI:** 10.1002/pola.28421

**Published:** 2016-11-21

**Authors:** C. Orofino, C. Foucher, F. Farrell, N. J. Findlay, B. Breig, A. L. Kanibolotsky, B. Guilhabert, F. Vilela, N. Laurand, M. D. Dawson, P. J. Skabara

**Affiliations:** ^1^ WestCHEM, Department of Pure and Applied Chemistry University of Strathclyde 295 Cathedral Street Glasgow G1 1XL UK; ^2^ Institute of Photonics, Department of Physics University of Strathclyde Glasgow G1 1RD; ^3^ Institute of Physical‐Organic Chemistry and Coal Chemistry Kyiv 02160 Ukraine; ^4^ School of Engineering and Physical Sciences, Chemical Sciences Heriot Watt University Edinburgh EH14 4AS UK

**Keywords:** Synthesis, oligomers, photophysics, aggregation induced emission, piezofluorochromism

## Abstract

A series of star‐shaped oligofluorene molecules, each containing a TPE core, have been specifically designed and produced to show effective aggregation‐induced emission (AIE). Each molecule differs either in the number of fluorene units within the arms (e.g., 1 or 4, compounds **4** and **5**), or the terminal group positioned at the end of each arm (e.g., H, TMS, or TPA, compounds **4**, **6**, and **7**). Although they are all poor emitters in solution phase they become efficient yellow‐green luminogens in the condensed state. Their AIE properties were investigated in THF/H_2_O mixtures, with each molecule exhibiting a clear emission enhancement at specific water contents. An all‐organic distributed feedback (DFB) laser was fabricated using compound **4** as the gain material and exhibited an average threshold energy fluence of 60 ± 6 μJ/cm^2^ and emission in the green region. Furthermore, piezofluorochromism studies on a thin film of this material displayed a linear dependence of the amplified spontaneous emission (ASE) peak position on applied pressure, indicating potential applications as lasing‐based pressure sensors. © 2016 The Authors. Journal of Polymer Science Part A: Polymer Chemistry Published by Wiley Periodicals, Inc. J. Polym. Sci., Part A: Polym. Chem. **2017**, *55*, 734–746

## INTRODUCTION

Many light‐emitting materials present efficient fluorescence in dilute solutions, while more concentrated solutions, or solid‐state samples, exhibit no emission. This is due to aggregation of chromophores *via* π–π stacking leading to formation of species that undergo decay via nonradiative pathways,^1^ commonly described as aggregation‐caused quenching (ACQ). Such an issue can be detrimental for application of organic materials in a series of optoelectronic and photonic devices such as organic light‐emitting diodes (OLED), lasers, and down‐converters, where the emissive layer is in the solid state. One of the ways to overcome the issues related to ACQ is to increase the dimensionality of the conjugated pathway in a fluorescent molecule.^2^ Macromolecular systems with extended dimensionality of the conjugated backbone, such as dendrimers and star‐shaped systems, are proven to be efficient fluorophores with high photoluminescence quantum yield (PLQY) both in solution and the solid state.^3^ The advantage of a star‐shaped macromolecular architecture of oligofluorene conjugated systems was demonstrated by the successful application of these materials in solid state down‐converters,^4^, ^5^ OLEDs,^6^ and organic lasers.^7^, ^8^ However, aggregation of star‐shaped conjugated systems cannot be completely avoided, especially for molecules with significant intermolecular charge transfer (ICT).^9^ Incorporating fluorophores into transparent polymer matrices, which is normally used for an intricate design of photonic devices,^10^, ^11^ can mitigate ACQ by spatial separation of the individual chromophores, but is not suitable for OLED fabrication.

In contrast to ACQ, aggregation induced emission (AIE), initially discovered for 1‐methyl‐1,2,3,4,5‐pentaphenylsilole by Ben Zhong Tang,^12^ relies on the formation of aggregated nanoparticles to enhance the emission of a material; all AIE‐active compounds are barely fluorescent in solution but become efficient emitters in the aggregated or solid state. Moreover, addition of varying fractions of water as a poor solvent to solutions of the compound in good organic solvents indicates a critical point at which fluorescence is greatly enhanced. The low fluorescence of AIE active materials in solution was postulated to be due to intramolecular rotations of the terminal phenyl substituents, which act as nonradiative relaxation pathways for excited state decay. Blocking these pathways by restricting the rotations in the aggregated or solid state lead to a decrease in fluorescence quenching, and the emission is dramatically enhanced.^13^ Among all AIE luminogens, tetraphenylethylene (TPE) combines the simplicity of its molecular structure with a strong AIE effect. The four phenyl rings are twisted (∼50°), adopting a propeller‐like conformation that avoids π stacking in the solid state while benefiting from further stabilization due to multiple C‐H–π bonds between the hydrogen atoms on the phenyl rings and the π‐electrons of the adjacent ring.^14^ Substituting the phenyl units with naphthyls in TPE does improve the thermal and electroluminescent properties of the resulting tetra‐β‐naphthylethene material but weakens its AIE effect.^15^ TPE has become a conventional hydrocarbon AIE unit, demonstrating enhanced emission in several non‐emissive materials, including the application of TPE as a side chain attached via flexible linkers in polyacteylene backbones, with fluorescence activated in the solid state.^16^ TPE has also been incorporated at the terminal position of oligofluorene, and the resulting materials exhibited an enhanced AIE depending on oligofluorene chain length; shorter chain analogs produced a more greatly enhanced AIE due to the higher proportion of TPE units present.^17^ Conjugated copolymers with different ratios of TPE and fluorene monomer units have been synthesized. The aggregation study of these copolymers with high contents of fluorene moieties in THF:water mixtures revealed the dual‐channel fluorescence response (DCFR), which was attributed to the AIE‐properties of TPE and AQE‐characteristics of the fluorene segments.^18^ Water‐soluble variants of these polymers were used for DCFR quantification of heparin.^19^ A thymidine‐functionalized AIE‐active TPE‐fluorene‐carbazole copolymer has been applied as the fluorescence turn‐on probe for aqueous Hg^2+^ solutions.^20^ The combination of TPE and triphenylamine (TPA) units led to the design of AIE‐active oligomers with good hole‐transporting properties and high PLQY, indicating their future role in the design of gain media for electrically pumped lasers.^21^

A series of star‐shaped materials containing the TPE unit are described in the literature, where the TPE structure is incorporated both in the arms and as the core of the molecule. A benzene‐cored star‐shaped material with pendant TPE arms exhibited AIE with a morphological change in the aggregates formed upon addition of water.^22^ At low water concentrations, crystalline aggregates were formed, while at high water concentrations, the aggregates were amorphous, resulting in a bathochromic shift of the emission and a decrease in intensity. Furthermore, the molecule showed selectivity for picric acid and Ru^3+^ ions, indicating an opportunity for sensing applications. Other examples employing TPE as the pendant groups with various cores have also been described.^23^, ^24^, ^25^

**Figure 1:**
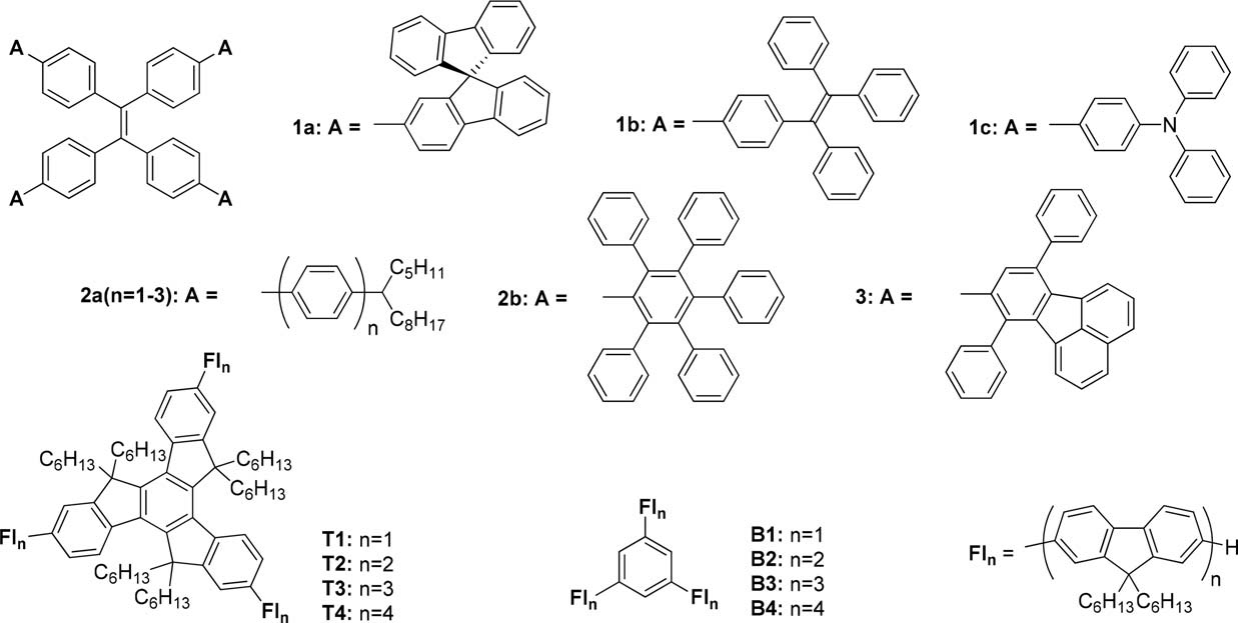
TPE‐cored star‐shaped systems **1–3** and trigonal oligofluorene series with truxene (**T1‐4**) and benzene (**B1‐4**) cores described in the literature.

TPE has also been incorporated as a core unit of star‐shaped compounds. Tang and coworkers disclosed a series of TPE‐cored molecules containing terminal groups consisting of either spiro[bifluorene] (**1a**), TPE (**1b**) or TPA (**1c**) arms (Figure 1).^26^ All exhibited weak fluorescence in solution but PLQYs of close to 100% in the solid state. Vyas and Rathore also described TPE‐cored analogs (**2a(*n* = 1–3)**) containing oligophenylene arm groups that suffered from a lack of emission through nonradiative decay pathways. Substitution with multiple phenyl rings introduced steric hindrance, blocking the nonradiative pathways, and significantly increasing the emission intensity of the final star‐shaped compound (**2b**).^27^, ^28^ More recently, the fluoranthene units have been incorporated in the arms of a TPE‐cored star‐shaped system (**3**), which has been tested as a fluorescent probe for the detection of nitroaromatic compounds (Figure 1).^29^

In contrast to the aforementioned macromolecular systems with extended dimensionality of their conjugated backbone, which proved to be efficient as gain media in the form of an amorphous film,^7^, ^8^, ^30^ AIE‐active systems have been mostly applied either as AIE‐dyes in cholesteric liquid crystal (CLC) gain materials^31^ or in single crystal lasing applications, where the AIE‐phenomenon offers an elegant way to provide high PLQY in a closely packed crystalline environment.^32^, ^34^

**Figure 2:**
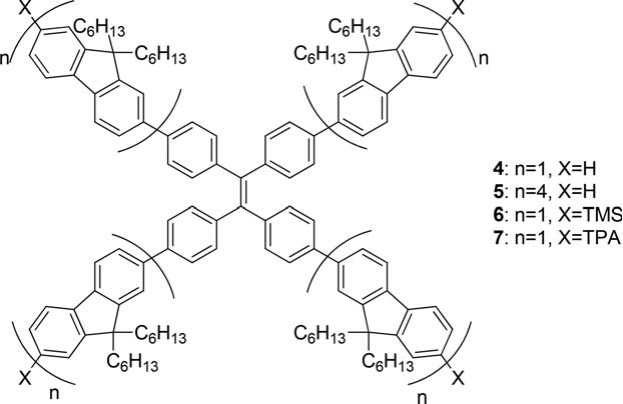
General structures of compounds **4**–**7**.

Herein, we discuss four molecular star‐shaped materials (**4**‐**7**), all containing the common tetraphenylethylene (TPE) core. Each differs either by the number of fluorene units constituting the pendant arm (1 or 4), or by the terminal end functionality (H, TMS or TPA) (Fig. 2).

The conjugated systems **4–7** were designed in order to combine the advantage of a star‐shaped macromolecular architecture with AIE‐properties endowed by a TPE core as new materials for lasing applications.

## EXPERIMENTAL

Dry solvents (dichloromethane, tetrahydrofuran, toluene, hexane and diethyl ether) were obtained from a solvent purification system (SPS 400, innovative technologies) using alumina as the drying agent; any other dry solvents were purchased from Sigma Aldrich. All reagents and solvents were purchased commercially from Sigma Aldrich or Alfa Aesar and were used without any purification. ^1^H and 13C NMR spectra were recorded on a Bruker Avance DPX400 (for compounds **5‐7**, **12** and for 13C NMR of compound **4**) at 400.13 and 100.61 MHz or a Bruker Avance DRX500 at 500 MHz(for ^1^H NMR of compound **4**) in CDCl_3_ or CD_2_Cl_2_. Proton NMR chemical shifts are reported as *δ* values in ppm relative to deuterated solvents: CDCl_3_ (7.26) or CD_2_Cl_2_ (5.32).^35^ Data are presented as follows: chemical shift, integration, multiplicity (s = singlet, b.s. = broad singlet, d = doublet, t = triplet, q = quartet, m = multiplet), and coupling constant(s) (*J*) are in Hz. Multiplets are reported over the range (in ppm) they appeared. Carbon NMR data were collected relative to the corresponding solvent signals CDCl_3_ (77.16) or CD_2_Cl_2_ (53.84).^35^ MS MALDI‐TOF spectra were recorded on a Shimadzu Axima‐CFR spectrometer (mass range 1‐150 000 Da). Elemental analyses were obtained on a PERKIN ELMER 2400 elemental analyzer. Compounds **8**, **9**, **10** and **11** were prepared according to literature procedures.^27^, ^36^, ^37^

UV‐Vis absorption spectra were recorded on a UNICAM UV 300, a Jasco V‐660 or a Shimazdu UV‐2600 spectrophotometer. Baselines of solvents were measured before analysis and solution spectra were recorded in 1 cm or 1 mm path length quartz cells between 190 and 900 nm. Emission spectra were measured on a Perkin Elmer LS45 or a Jasco FP‐6500 fluorescence spectrometers. Cyclic voltammetry measurements were performed on a CH Instruments 660A electrochemical workstation with *iR* compensation using anhydrous solvents (dichloromethane or 1:2 acetonitrile:benzene). The electrodes were glassy carbon, platinum wire, and silver wire as the working, counter, and reference electrodes, respectively. All solutions were degassed (Ar) and contained compounds in concentrations *ca*. 10^−4^ M, together with TBAPF_6_ (0.1 M) as the supporting electrolyte. All measurements are referenced against the *E*_1/2_ of the Fc/Fc^+^ redox couple. Thermogravimetric analysis was performed using a Perkin Elmer Thermogravimetric Analyser TGA7 under a constant flow of helium (20 mL/min). The percentage weight loss over time was recorded at this temperature and the data was processed using the Pyris Series Software. Differential scanning calorimetry was conducted on a TA Instruments Q1000 with a RC‐90 refrigerated cooling unit attached. The calibration was conducted using indium (melt temperature 156.42 °C, ΔHf 28.42 J/g).

Amplified spontaneous emission (ASE) studies were carried out in air by exciting the structures using a frequency‐tripled Nd:YAG laser (Continuum Minilite), which produced pulses of 10 ns duration (FWHM) with a repetition rate of 10 Hz at *λ* = 355 nm. The pump beam was shaped into a 3 mm × 0.3 mm stripe on the surface of the structures under test. The latter consisted of films of the TPE‐core molecules formed by spin‐coating onto unpatterned NOA65 epoxy substrates. A half‐waveplate/polarizer combination was used to control the energy of the pump light incident on the samples. The edge emission of the structures was coupled into a CCD spectrometer (maximum resolution of 0.13 nm) through a 50‐μm core optical fiber, and the optical spectrum was recorded at different pump energies. The fiber was placed transverse to the edge of the pump stripe, at the end of the sample film, to collect the wave‐guided emission.

Characterization of the second‐order DFB lasers was similar than for the ASE measurements but the detection of the laser output was collected in the direction vertical to the plane of the film structures. The DFB lasers were fabricated by forming a 110 ± 10‐nm‐thick film of TPE molecules onto a surface‐patterned epoxy substrate. The periodicity of the pattern was chosen so the structure has a second‐order DFB cavity, leading to a vertical laser output above the oscillation threshold.

### Compound 4

A mixture of tetra‐4‐bromophenylethene (**8**) (75 mg, 1.16 × 10^−4^ mol, 1.0 eq), Pd(PPh_3_)_4_ (53 mg, 4.63 × 10^−5^ mol, 0.4 eq), (9,9‐dihexyl‐9H‐fluoren‐2‐yl)boronic acid (**9**) (278 mg, 7.36 × 10^−4^ mol, 6.4 eq) and Ba(OH)_2_·8H_2_O (366 mg, 1.13·10^−3^ mol, 9.6 eq.) was dissolved in anhydrous THF (20 mL). The solution was degassed with N_2_, followed by the addition of water (0.6 mL) and the system was stirred under N_2_ at 70 °C for 18 h. The reaction was quenched with an aqueous solution of NH_4_Cl (50 mL) and extracted with dichloromethane (4 × 50 mL). The combined organic fractions were washed with water (100 mL), dried over anhydrous MgSO_4_ and the solvent evaporated to yield a dark green oil. The crude product was purified by column chromatography on silica gel eluting with hexane/dichloromethane (9:1). The material was dissolved in the minimum amount of dichloromethane and re‐precipitated from methanol to yield the product (**4**) as a bright yellow powder with *T*_g_ = 82 °C and *T*_d_ (5% mass loss) = 439 °C (153 mg, 9.21 × 10^−5^ mol, 80%); ^1^H NMR (CD_2_Cl_2_) *δ* (ppm): 7.75 (4H, d, 3 *J* = 7.8 Hz), 7.73–7.68 (4 H, m), 7.61 (4H, s), 7.60 (4H, dd, 3 *J* = 7.8 Hz, 4 *J* = 1.6 Hz), 7.57 (8H, d 3 *J* = 8.4 Hz), 7.38–7.25 (12 H, m), 7.29 (8H, d, 3 *J* = 8.4 Hz), 2.06–1.93 (16 H, m), 1.18–0.97 (48 H, m), 0.74 (24 H, t, 3 *J* = 7.2 Hz), 0.69–0.50 (16 H, m); 13C NMR (CD_2_Cl_2_) *δ* (ppm): 151.86, 151.39, 143.42, 141.14, 140.93, 140.76, 139.90, 139.65, 132.38, 127.45, 127.15, 126.73, 126.03, 123.32, 121.52, 120.27, 120.04, 55.57, 40.76, 31.90, 30.05, 24.18, 22.95, 14.16; (MALDI/TOF, m/z): [M^+^] calcd. for C_426_H_532_: 1662.5; found, 1661.21; Anal. calcd. for C_126_H_148_: C, 91.03; H, 8.97;. Found: C, 90.66; H, 9.30.

### Compound 5

A mixture of tetra‐4‐bromophenylethene (**8**) (69.8 mg, 1.08 × 10^−4^ mol, 1.0 eq.), Pd(PPh_3_)_4_ (50 mg, 4.31 × 10^−5^ mol, 0.4 eq), (9,9,9’,9’,9’’,9’’,9’’’,9’’’‐octahexyl‐9H,9'H,9”H,9””H‐[2,2’:7’,2”:7”,2””‐quarterfluoren]‐7‐yl)boronic acid (**10**) (960 mg, 6.85 × 10^−4^ mol, 6.4 eq.) and Ba(OH)_2_·8H_2_O (341 mg, 1.05 × 10^−3^ mol, 9.6 eq.) was dissolved in anhydrous THF (20 mL). The solution was degassed with N_2_, followed by the addition of water (0.6 mL) and the system was stirred under N_2_ at 70 °C for 18 h. The reaction was quenched with an aqueous solution of NH_4_Cl (50 mL) and extracted with dichloromethane (4 × 50 mL). The combined organic fractions were washed with water (100 mL), dried over anhydrous MgSO_4_ and the solvent evaporated to yield a dark green oil. The crude product was purified by column chromatography on silica gel eluting with petroleum ether:toluene (9:1) stabilized with triethylamine (1%). The material was dissolved in the minimum amount of dichloromethane and re‐precipitated from methanol to yield the product (**2**) as a bright yellow solid with *T*_g_ = 105 °C and *T*_d_ (5% mass loss) = 470 °C (549 mg, 9.72 × 10^−5^ mol, 90%). ^1^H NMR (CDCl_3_) *δ* (ppm): 7.90–7.55 (100 H, m), 7.40–7.30 (16 H, m), 2.25–1.95 (64 H, m), 1.20–1.00 (192 H, m), 0.90–0.65 (160 H, m). 13C NMR (CDCl_3_) *δ* (ppm): 151.94, 151.61, 151.14, 140.93, 140.67, 140.63, 140.46, 140.32, 140.15, 139.64, 139.53, 132.27, 127.15, 126.92, 126.60, 126.29, 126.17, 126.05, 123.07, 121.65, 121.57, 121.27, 120.12, 120.03, 119.86, 55.46, 55.30, 40.51, 31.62, 31.60, 29.84, 29.81, 23.97, 23.92, 22.73, 22.70, 14.17. (MALDI/TOF, m/z): [M^+^] calcd. for C_426_H_532_: 5652.8; found, 5652.2. Anal. calcd. for C_426_H_532_: C, 90.51; H, 9.49;. Found: C, 90.00; H, 9.89.

### Compound 6

Compound **8** (0.71 g, 1.09 mmol, 1.0 eq.) was added to a flask containing (9,9‐dihexyl‐7‐(trimethylsilyl)−9H‐fluoren‐2‐yl)boronic acid pinacol ester **11** (3.71 g, 6.96 mmol, 6.4 eq.), barium hydroxide (3.30 g, 10.46 mmol, 9.6 eq.) and tetrakis(triphenylphosphine) palladium(0) (0.50 g, 0.44 mmol, 0.4 eq.). The mixture was purged with Ar and tetrahydrofuran (60 ml) was added. Degassed water (6.6 mL) was added to solubilize the barium hydroxide followed by tetrahydrofuran (10 mL) to rinse the condenser. The reaction was purged with Ar after each solvent addition (×5) and stirred at 65 °C for 4 days. The reaction was quenched with saturated ammonium chloride (200 ml) and extracted with dichloromethane (3 × 100 mL). The organic layers were combined, dried using magnesium sulfate and concentrated under vacuum. The product was separated from by‐products and starting material via silica column chromatography, with hexane and dichloromethane (9:1). The isolated product was concentrated and dried to obtain a yellow/green solid. This was dissolved in a minimal amount of hot dichloromethane and re‐precipitated with methanol until a powdered solid remained in the mixture. The product was stored in the freezer overnight, then filtered and washed using cold methanol to afford compound **6** as a yellow solid with *T*_g_ = 109 °C and *T*_d_ (5% mass loss) = 411 °C (1.25 g, 56%); ^1^H NMR (CDCl_3_) *δ*: 7.73 (4H, d, 3 *J* = 8.3 Hz), 7.68 (4H, d, 3 *J* =7.5 Hz), 7.72–7.56 (8H, m), 7.53 (8H, d, 3 *J* = 8.4 Hz), 4.49 (4H, dd, 3 *J* = 7.5 Hz, 4 *J* = 0.7 Hz), 7.46 (4H, s), 7.28 (8H, d, *J* = 8.4 Hz), 2.10–1.90 (16H, m), 1.20–0.95 (48H, m), 0.85–0.55 (40H, m), 0.31 (36H, s); 13C NMR (CDCl_3_) *δ*: 13C NMR (101 MHz, CDCl_3_) δ 151.79, 150.28, 143.08, 141.56, 139.67, 139.14, 132.21, 127.74, 126.57, 125.42, 121.28, 120.14, 119.14, 55.24, 40.33, 31.49, 29.72, 23.82, 22.63, 14.13, −0.73.; *m/z* (MALDI) 1952.12 [(M‐H)+ 100%], 1950.12 (M‐H)‐; Anal. Calculated: (%) C, 84.94, H, 9.30; Found (%) C, 85.19, H, 8.90.

### Compound 12

Compound **6** (232 mg, 0.12 mmol, 1.0 eq.) and sodium acetate (39 mg, 0.48 mmol, 4.0 eq.) were added to a reaction flask, evacuated and purged with Ar. Anhydrous THF (14 mL) was added, the solution cooled to 0 °C and covered to avoid light exposure. A solution of bromine (177 mg, 1.11 mmol, 9.32 eq.) in dichloromethane (1 mL) was added and the mixture stirred for 17 h. The reaction was quenched with triethylamine (0.31 mL, 2.2163 mmol, 18.64 eq.), followed by aqueous sodium sulfite (65 mL) and extracted with dichloromethane (100 mL, 2 × 25 mL). The combined extracts were then washed with sodium bicarbonate (75 mL) and dried with MgSO_4_. The resultant yellow solid was dissolved in hexane and filtered through a plug of silica using hexane and dichloromethane (1:1), to afford the target compound (**9**) as a yellow solid (221 mg, 94%), which was used in the next step without further purification; ^1^H NMR (CDCl_3_) *δ*: 7.68 (4H, d, 3 *J* = 7.9 Hz), 7.61–7.53 (12H, m), 7.52 (8H, d, 3 *J* =8.4 Hz), 7.45 (4H, d, 4 *J* = 1.8 Hz), 7.45 (4H, dd, 3 *J* = 8.4 Hz, 4 *J* = 1.7 Hz), 7.27 (8H, d 3 *J* = 8.4 Hz), 2.02–1.87 (16H, m), 1.15–0.96 (48H, m), 0.80–0.70 (24H, m), 0.67–0.55 (16H, m).

### Compound 7

#### Part (I)

4‐Diphenylaminophenyl boronic acid (187 mg, 0.65 mmol, 6.4 eq.), tetrakis(triphenyl‐phosphine) palladium (0) (46.7 mg, 0.04 mmol, 0.4 eq.) and barium hydroxide (306 mg, 0.97 mmol, 9.6 eq.) were charged to a flask containing **12** (200 mg, 0.10 mmol, 1.0 eq.). The mixture was purged with Ar and anhydrous tetrahydrofuran was added (8 mL); the mixture was purged again and degassed water (0.61 mL) was added. The reaction was purged and tetrahydrofuran (2 mL) was added to rinse the condenser. The resultant orange solution was purged (x5) and left stirring at 65 °C for 24 h. The reaction was quenched with saturated ammonium chloride (50 mL) and extracted with dichloromethane (3 × 50 mL). The organic layers were combined, dried with magnesium sulfate and concentrated. The sample was absorbed onto silica and silica gel column chromatography was carried out using 20% dichloromethane in hexane. The impure product was isolated and concentrated to obtain a yellow gum (170 mg).

#### Part (II)

4‐Diphenylaminophenyl boronic acid (93.5 mg, 0.324 mmol, 6.4 eq.), tetrakis(triphenyl phosphine) palladium (0) (23.4 mg, 0.02 mmol, 0.4 eq.) and barium hydroxide (153 mg, 0.49 mmol, 9.6 eq.) were charged to a microwave vessel containing **12** (100 mg, 0.051 mmol, 1.0 eq.). The mixture was purged with Ar and anhydrous tetrahydrofuran was added (3.5 mL), the mixture was purged again and degassed water (0.3 mL) was added. The resultant orange solution was purged (x5) and placed in the microwave at 80 °C for 2 h. The reaction was quenched with saturated ammonium chloride (20 mL) and extracted with dichloromethane (2 × 25 mL); the organic layers were combined and dried over magnesium sulfate. The yellow solid was dissolved in hexane and filtered through a plug of silica to afford a light yellow residue.

#### Part (III)

The combined products of parts (i) and (ii) were absorbed onto silica and silica gel column chromatography was carried out using 20% dichloromethane in hexane. The product fraction was isolated, concentrated and dried under vacuum. The product was dissolved in a minimal amount of hot dichloromethane then precipitated using cold methanol and stored in the freezer for 1 h. The resultant yellow solid with *T*_g_ = 140 °C and *T*_d_ (5% mass loss) = 433 °C was filtered through a sinter funnel, washed with cold methanol before being dried under vacuum and collected to afford compound **7** (110 mg, 28%); ^1^H NMR (CDCl_3_) *δ*: 7.73 (8H, d, *J* = 7.8 Hz), 7.62–7.51 (32H, m), 7.32–7.24 (24H, m), 7.20–7.11 (24H, m), 7.04 (8H, tt, 3 *J* = 7.3 Hz, 4 *J* = 2.2 Hz), 2.07–1.96 (16H, m), 1.15–0.98 (48H, m), 0.77–0.63 (40H, m); 13C NMR (CDCl_3_) *δ*: 13C NMR (101 MHz, CDCl_3_) δ 151.81, 147.87, 147.22, 143.10, 140.36, 139.84, 139.64, 139.43, 135.82, 132.23, 129.43, 127.94, 126.57, 126.00, 125.69, 124.52, 124.20, 123.06, 121.21, 121.09, 120.11, 120.04, 55.41, 40.66, 31.62, 29.86, 23.93, 22.73, 14.14.; *m/z* (MALDI): 2635.38 [(M) 100%], 2636.44 (M‐H)+, 2633.41 (M‐2H)‐; Anal. Calculated (%): C, 90.23, H, 7.65, N, 2.13; Found (%): C, 89.94, H, 7.55, N, 2.17.

## RESULTS AND DISCUSSION

### Synthesis

**Scheme 1:**
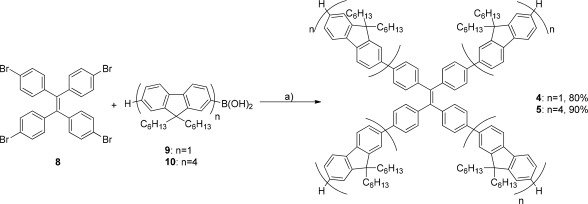
Synthesis of oligofluorene TPE analogs **4** and **5**. Reagents and conditions: (a) Pd(PPh_3_)_4_, Ba(OH)_2_.8H_2_O, THF/H_2_O (10:1), reflux, 18 h.

The syntheses of monofluorene and quaterfluorene analogs **4** and **5** are detailed in Scheme 1. Both materials were obtained in high yields (80% for compound **4**, 90% for **5**) *via* Suzuki coupling conditions employing barium hydroxide as base and Pd[PPh_3_]_4_ as catalyst, with tetrabromo‐TPE (**8**) and either monofluorene (**9**) or quaterfluorene (**10**) boronic acids.^36^, ^37^

**Scheme 2:**
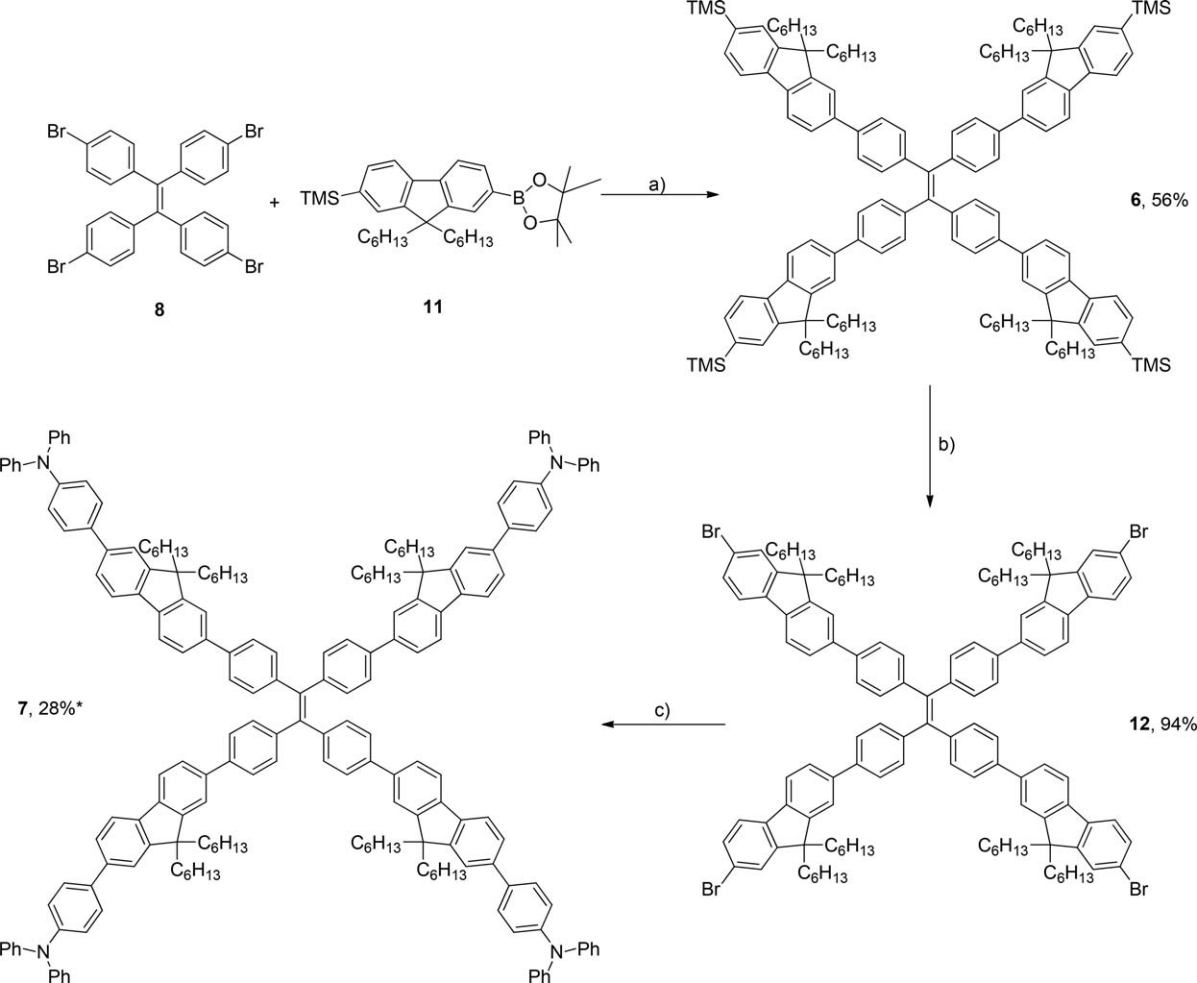
Synthesis of TMS and TPA‐capped fluorene TPE analogues **6** and **7**. Reagents and conditions: a) Pd(PPh_3_)_4_, Ba(OH)_2_.8H_2_O, THF/H_2_O (10:1), reflux, 18 h; b) Br_2_, NaOAc, THF, 17 h; c) 4‐(diphenylamino)phenyl boronic acid, Pd(PPh_3_)_4_, Ba(OH)_2_.8H_2_O, THF/H_2_O (10:1), reflux, 24 h *and* 4‐(diphenylamino)phenyl boronic acid, Pd(PPh_3_)_4_, Ba(OH)_2_.8H_2_O, THF/H_2_O (10:1), microwave, 2 h. * The yield of 28% is derived from the combination of both sets of conditions and further purification.

The syntheses of compounds **6** and **7** were also relatively straightforward (Scheme 2). Suzuki conditions, using TMS‐protected monofluorene boronic ester (**11**), affords compound **6** in moderate yield. Conversion of the terminal TMS groups using bromine yielded intermediate **12** in high yield, allowing a further Suzuki coupling with 4‐(diphenylamino)phenyl boronic acid to afford **7**. Conversion to compound **7** was low using both conventional and microwave conditions, hence both crude reaction mixtures were combined and purified as one. All four compounds were obtained as bright yellow powders in high purity.

### Physical Characterization

#### Thermal Properties

Thermal gravimetric analysis (TGA) revealed good thermal stability of compounds **4–7** with decomposition temperatures (*T*_d_, 5% mass loss) above 400 °C in inert atmosphere (Table 1, Supporting Information Fig. S1). The least stable was the TMS‐functionalized tetrakis(4‐(dihexyl‐fluoren‐2‐yl)phenyl)ethene **6**, with a *T*_d_ of 411 °C. The star‐shaped oligomer **5** with the longest quarterfluorenyl arms exhibited the highest decomposition temperature of 470 °C, with oligomers **4** and **7** showing similar *T*_d_ in the range of 430–440 °C.

**Table 1:** Thermal Properties of Compounds **4–7**

Compound	*M*_w_ (g·mol^−1^)	*T*_g_ (°C)	*T*_d_ [TGA, 5% mass loss] (°C)
**4**	1,662.52	82	439
**5**	5,652.78	105	470
**6**	1,951.25	109	439
**7**	2,635.73	140	433

TPE derivatives **4–7** are amorphous materials with glass transition temperatures (*T*_g_) ranging from 82 to 140 °C (Supporting Information Fig. S2). Of all the compounds in this series, the simplest star‐shaped system **4** shows the lowest *T*_g_ of 82 ^o^C, which is still almost 20 °C higher than that of the first member of the oligofluorene‐truxene series (**T1**).^36^ Extending the arms up to four fluorene units and the addition of the TMS functionalities at the terminal positions of compound **4** led to an increase of 23–27 °C for *T*_g_ of compound **5** and **6**. The highest glass transition temperature was registered for compound **7** bearing four TPA units.

#### Electrochemistry

The electrochemical properties of compounds **4–7** are summarized in Table 2. The cyclic voltammograms (CV) of compounds **4–7** (Supporting Information Fig. S3) were run in an acetonitrile: benzene (1:2) mixture up to the highest potential of 1.2–1.3 V versus Fc/Fc^+^. Extending the potential range further led to over‐oxidation for all the compounds with no discernible peaks, but shoulders registered. The TPA containing oligomer **7** exhibited three reversible waves upon oxidation, the first one ( $E_{\text{Ox}}^{1/2}$ = 0.54 V) being related to the oxidation of the four TPA units. The waves at 0.86 V and 1.03 V are due to the consecutive oxidation of the central tetrakis(4‐(fluoren‐2‐yl)phenyl)ethene unit, which is consistent with the electrochemical behavior of TPE‐cored star‐shaped oligophenylenes.^27^ The CVs of compounds **4** and **6** show only one reversible wave at around 0.8 V. In the case of compound **5**, this wave overlaps with at least two other oxidation waves related to a further oxidation of the quaterfluorene arms. In dichloromethane, the oxidation of compound **7** (Supporting Information Fig. S4) is very similar to that in the acetonitrile: benzene mixture, with a very close positioning of the second and third oxidation waves and significantly lower oxidation potentials due to better solvation of the cation‐radical. It is interesting to note that compound **6** in CH_2_Cl_2_ exhibits upon oxidation two very closely positioned waves (Supporting Information Fig. S4), which allows us to assume that the single reversible oxidation wave observed in the CVs of compounds **4** and **6** in acetonitrile: benzene mixture is, in fact, two nonresolved one‐electron oxidations of the tetrakis(4‐(fluoren‐2‐yl)phenyl)ethene unit. In the acetonitrile: benzene mixture, all the compounds exhibit a single quasi‐reversible (irreversible in the case of **5**) reduction wave with the cathodic peak positioned at 2.3 ‐ 2.5 V.

**Table 2:** Electrochemical Properties of Compounds **4–7**

Compound	$E_{\text{ox}}^{p}$ (V)^a^	HOMO (eV)^c^	$E_{\text{red}}^{p}$ (V)^a^	LUMO (eV)^c^	$E_{g}^{\text{EC}}$ (eV)	$E_{g}^{\text{opt}}$ (eV)^e^
**4**	0.86/0.77	−5.54	−2.46/‐2.20	−2.50	3.04	2.96
**5**	1.01/0.85^b^	−5.54	−2.44^d^	−2.53	3.01	3.00
**6**	0.85/0.73	−5.50	−2.54/‐2.46	−2.43	3.07	2.96
**7**	0.56/0.51; 0.91/0.82; 1.05/1.01	−5.27	−2.34/‐2.23	−2.58	2.69	2.96

a The positions of peaks on forward/reversed scans are given vs the Fc/Fc^+^ redox couple.

b Only the resolved peaks are presented among the overlapping waves.

c HOMO/LUMO values were calculated using the formula HOMO/LUMO = − $E_{\text{ox}}^{\text{onset}}$/ $E_{\text{red}}^{\text{onset}}$ − 4.80.

d The cathodic peak of the irreversible reduction wave is presented.

e The optical HOMO‐LUMO gap was estimated using the formula $E_{g}^{\text{opt}}$ = 1239.84/*λ*_onset_.

#### Optical Properties

**Figure 3:**
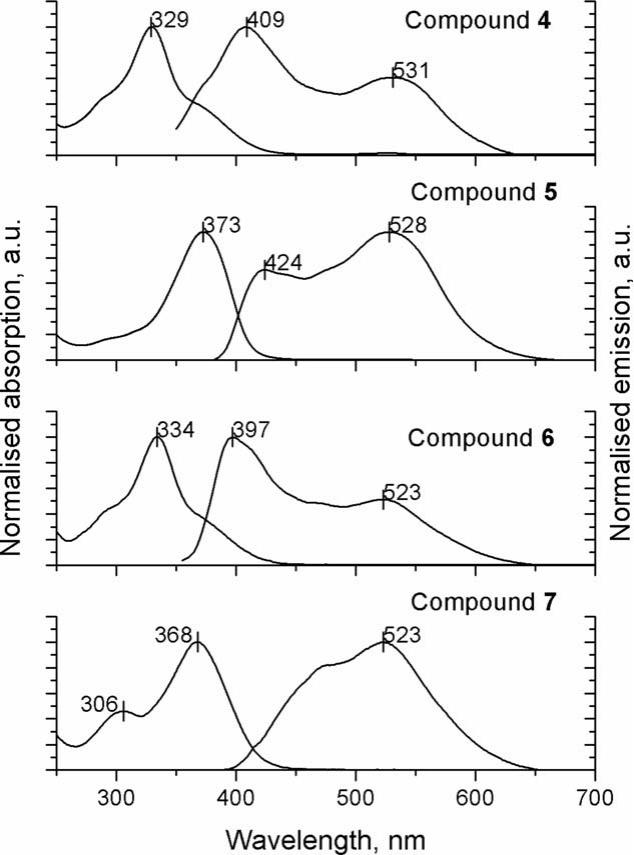
Normalized absorption and PL spectra of compounds **4–7** in dichloromethane. *λ*_ex_ (compound **4**) = 329 nm, *λ*_ex_ (compound **5**) = 372 nm, *λ*_ex_ (compound **6**) = 334 nm, *λ*_ex_ (compound **7**) = 368 nm. The peak values of the absorption/ emission spectra are marked.

The absorption and emission properties in dichloromethane for all four compounds **4–7** are presented in Figure 3. Compounds **4** and **6** revealed an absorption band with a maximum at ∼ 330 nm along with two shoulders at short‐wave (∼280 nm) and long‐wave (∼370 nm) regions. The low wavelength feature can be assigned to the absorption of the TPE core^38^, whereas the band with a peak at ∼330 nm is related to the fluorenyl‐phenyl unit, with the position of the peak close to that of the absorption spectrum of tris(fluorenyl)benzene (**B1**).^39^, ^40^ The long‐wave shoulder is due to the transition from the HOMO which extends from the core to the whole molecule. Similar features have been observed in the absorption spectra of the star‐shaped structure with a TPE core and biphenylene arms (**2a(n = 2)**) (Figure 1).^27^

The spectrum of compound **5** reveals a featureless absorption peak at 373 nm, probably dominated by the π–π* transition of the much longer quaterfluorenyl‐phenyl arms. On the other hand, compound **7** exhibits two absorption bands with maxima at 306 and 368 nm. The first can be related to the TPE core, while the other more intense band, as in the case of compound **5**, comprises of the absorption of TPE‐decorated arms and the transition from the molecular orbital which extends from the TPE core to the fluorene units. As mentioned above, this orbital is likely to be the HOMO in the star‐shaped systems **4**‐**6**. Previously it was found for the TPE cored terphenylene star‐shaped oligomer **2a(n = 3)** that its HOMO would reside on the TPE core and only the first two phenylene units of the arms.^27^

Investigation of the UV‐Vis absorption of compounds **4–5** in various solvents, including the 1:2 acetonitrile:benzene mixture, dichloromethane, tetrahydrofuran and hexane did not reveal any significant solvatochromism (Table 3, Supporting Information Fig. S5), with the spectra in hexane showing a slight hypsochromic shift, as the less polar solvent can increase the energy of the π* level. It was found that the shape of the spectra in all of these solvents did not depend on the concentration in the range of *C* = 10^−7^ – 10^−4^ M, with the optical properties in an acetonitrile: benzene mixture and dichloromethane being very similar.

**Table 3:** UV–Vis Absorption Properties of Compounds **4–5** in Different Solvents and Compounds **6‐7** in CH_2_Cl_2_

	Peak Maximum (nm) [log ε]/Absorption Onset (nm)			
Compound	AN:Benzene	CH_2_Cl_2_	Hexane	THF
**4**	330 (5.03)/419	329 (5.08)/419	327 (4.98)/417	331 (5.12)/420
**5**	372 (5.72)/413	373 (5.76)/413	368 (5.79)/410	374 (5.72)/414
	Peak maximum (nm) [log ε]/Absorption onset (nm) in CH_2_Cl_2_			
**6**	334 (5.25)/419			
**7**	306 (5.15), 368 (5.49)/420			

In solution, the emission properties of compounds **4–7** are poor due to nonradiative decay associated with the rotation of arms around the ethene core. The fluorescence spectra (Fig. 3) of compound **7** at ∼10^−7^ M revealed a peak at 523 nm with a shoulder in the short‐wave region. In contrast, for compounds **4‐6**, two emission bands were registered, one around 400 nm associated with the emission of the arms, while the second one appears around 530 nm and is related to the emission of the whole molecule for compounds **4** and **6** and the central part of molecule **5** (which includes the TPE core and one fluorene unit from each arm). The relative intensity of these two bands was found to depend on the concentration of the solutions. Supporting Information Figure S6 presents the fluorescence spectra of compounds **4–5** at different concentrations in CH_2_Cl_2_ and hexane solutions. At low concentrations (up to ∼10^−6^ M, for compound **4** in CH_2_Cl_2_) the short‐wave band dominates the spectrum and, as the concentration of the materials increases, the intensity of this band diminishes and the long‐wave band becomes dominant (at around 6 × 10^−5^ M). This might be a consequence of a greater degree of aggregation of the molecules in concentrated solutions. However, self‐absorption could be partially responsible for the change in the luminescence spectra at higher luminophore concentration.^41^

In contrast to the aforementioned hypsochromic shift in the absorption spectra, in hexane the emission occurs at slightly longer wavelengths for both compounds **4** and **5**. A similar observation was made for the linear oligofluorene TPE compounds, with an increased planarization of the TPE‐containing molecules in the excited state in hexane solution being assumed.^17^ In the case of compounds **4** and **5**, the planarization of the excited state due to restricted rotation in hexane is also consistent with the less pronounced emission at ∼400 nm in this solvent compared to measurements in dichloromethane.

**Figure 4:**
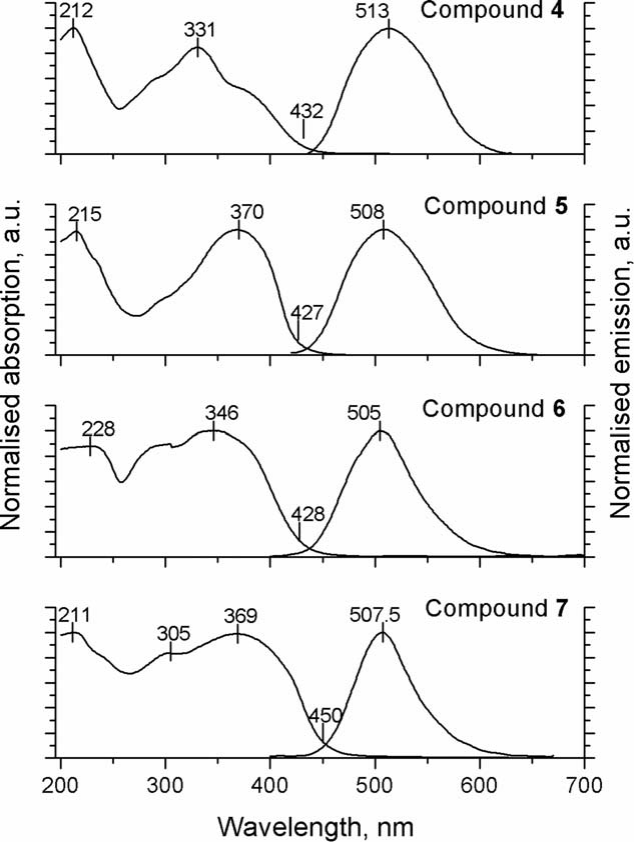
Normalized absorption and PL spectra of compounds **4–7** in the solid state as films. *λ*_ex_ (compound **4**) = 330 nm, *λ*_ex_ (compound **5**) = 370 nm, *λ*_ex_ (compound **6**) = 349 nm, *λ*_ex_ (compound **7**) = 368 nm. The peak values of absorption/emission spectra and the onset of absorption are marked.

The absorption and emission spectra of the materials in the films drop‐cast from 20 mg·mL^−1^ solutions in toluene are presented in Figure 4. The profiles of the absorption bands follow the same pattern as in solution but are slightly broader. The position of the absorption peak does not change significantly but the relative intensities of long and short‐wave shoulders for compounds **4** and **6** increase compared to that of the central band. This increase is particularly pronounced for compound **6**, which makes the absorption peak very broad and leads to an apparent bathochromic shift of *λ*_max_. Interestingly, the absorption spectra of compound **5** and **7**, while maintaining the position of the main peak, reveal a shoulder‐like appearance at the long‐wave region of the band. All these changes red‐shift the absorption onset of all four compounds significantly.

The emission profile does not show any hint of fluorescence from the isolated arms and only one band is revealed for compounds **4–7**. The position of this band is hypsochromically shifted for all the compounds with respect to the solution emission profile. The aforementioned bathochromic shift of absorption onset suggests a more rigid and planar structure of the ground state. However, the structural relaxation of the excited state is sterically hindered in the solid phase, which leads to a hypsochromic shift of the emission. The PLQYs were determined from powders of materials **4** (29%) and **5** (77%) and from films for compounds **6** (85%) and **7** (84%). The emission is dramatically enhanced for all compounds in the condensed state due to the restriction of intramolecular rotations (RIR) effect and the molecules are therefore AIE active.

#### Aggregation Induced Emission in Water: THF Mixtures

**Figure 5:**
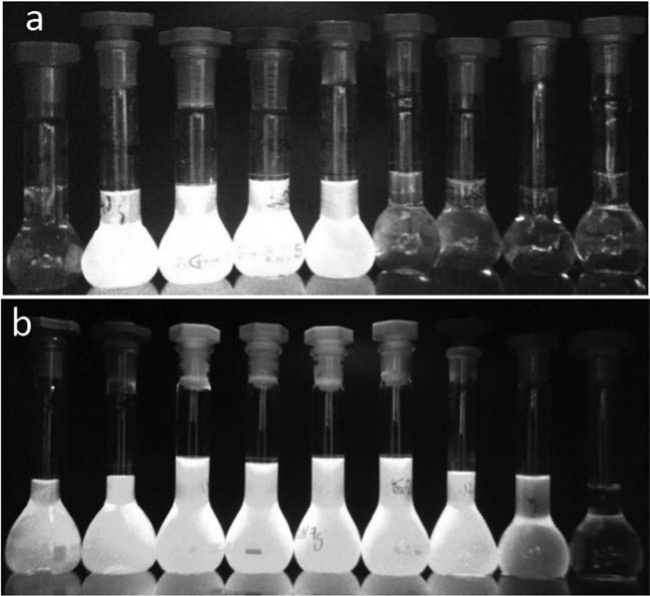
10 μM solutions of **4** (a) and **5** (b) in THF:H_2_O mixtures with increasing water fractions under UV (365 nm) illumination. From right to left 10, 20, 30, 40, 50, 60, 70, 80, and 90% water contents.

In order to compare directly the emissive properties of TPE‐cored star‐shaped oligofluorenes in solution and in the aggregated state, the PL spectra of compounds **4**, **5** and **7** were studied in THF solutions with varying fractions of water. The most prominent changes in the emission of compounds **4** and **5** were observed for their 10 µM solutions. The AIE effect can be visually observed, and Figure 5 presents photos of a series of 10 µM solutions in THF:H_2_O mixtures with different amounts of water for compounds **4** [Fig. 5(a)] and **5** [Fig. 5(b)]. The solutions of the materials with increasing water fractions are placed from right to left. The colored photos are shown in the Supporting Information Figure S7a,b.

The solutions of **4** in THF are barely emissive and their fluorescence is only boosted when 50% of water is added to the solvent mixture. The fluorescence becomes brighter up to 80% water content and then decreases at 90%. Compound **5** also shows an enhancement of its emission that can be observed with only a 20% water fraction, becoming brighter and greener (rather than yellow) at 30–70% water content, before it decreases again upon the addition of greater amounts of water to the mixture. The solutions exhibiting AIE show some turbidity, especially for compound **5**, but no precipitation or obvious agglomerates can be observed with the naked eye. The effect is probably due to the formation of nano‐aggregates in which the AIE active molecules are forced to be close to each other, restricting the intramolecular rotations and enhancing PL upon the addition of water.

The absorption spectra of 10^−5^ M solutions of **4** in different water:THF mixtures is presented in Supporting Information Figure S7c. The spectra of the solutions with up to 40% water content do not change compared to the pure THF solution. Nevertheless, adding 50% of water to the mixture triggers a sudden lowering in the absorption intensity and broadening of the profile (so the area of absorption actually increases), accompanied by a bathochromic shift of all the peaks and an increase in the intensity of the shoulder located at the long‐wave edge of the absorption maximum. Moreover, the absorption profile levels off from the baseline at longer wavelengths due to possible light scattering. All of these effects are an indication of the formation of nano‐aggregates in solution.

**Figure 6:**
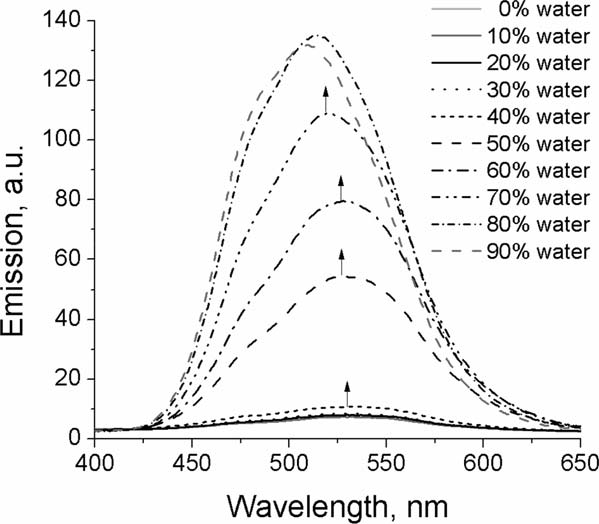
Emission spectra of 10^−5^ M solutions of **4** in THF:H_2_O mixtures with increasing water fraction. *λ*_ex_ = 330 nm.

The emission spectra of the same solutions (Fig. 6) are very weak (10 a.u.) up to the addition of 40% of water to the mixture, but they increase in intensity with greater water content, achieving a maximum at 80%. The emission maxima suffer a slight hypsochromic shift with the increase of the water content, which could be due to the formation of aggregates in which the structural relaxation of the excited state is more sterically hindered due to spatial constraint. The fact that the emission of the aggregates does not suffer bathochromic shifts could be an indication that the molecules do not form excimers through π–π stacking, which typically show red‐shifted emissions.

The absorption spectra of 10^−5^ M solutions of **5** in water:THF mixtures with increasing water fractions are presented in Supporting Information Figure S8. In this case, the addition of 20% water is enough to trigger the nano‐aggregate formation, which is again accompanied by the scattering signature, a decrease in absorption intensity and a broadening of the absorption profile. The larger size of molecule **5**, in comparison to that of **4**, and its more hydrophobic nature (32 hexyl chains in compound **5** versus 8 in **4**), lead to a lower threshold of water that triggers the formation of the aggregated clusters in this case.

**Figure 7:**
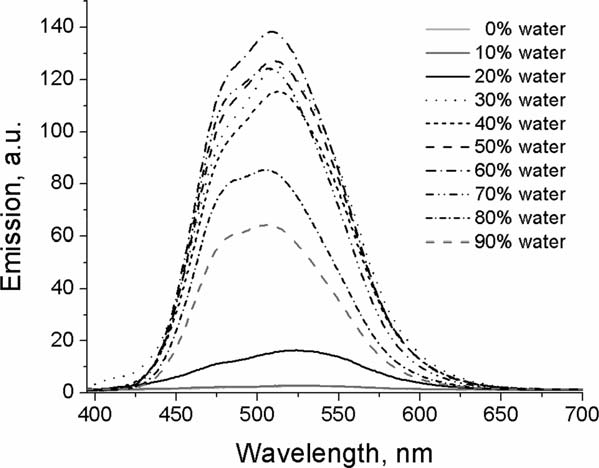
Emission spectra of 10^−5^ M solutions of **5** in THF:H_2_O mixtures with increasing water fractions. *λ*_ex_ = 370 nm.

The emission spectra of the same solutions of compound **5** are shown in Figure 7. Only 20% of water is enough to increase the fluorescence of the solution, but the addition of 30% of water triggers a great leap in the emission intensity. When the absorption and emission spectra of solutions aged for one month were recorded, there was little variation in their profiles or intensities, indicating that the particles that are formed are stable over this period of time. Similar PL measurements of solutions in THF:H_2_O mixtures were performed for compound **7** (Supporting Information Fig. S9), which exhibited significant enhancement of emission after addition of 40% of water. The emission, in this case, continues to grow up to a water content of 90%, with a bathochromic shift revealed after reaching 70% water content.

**Figure 8:**
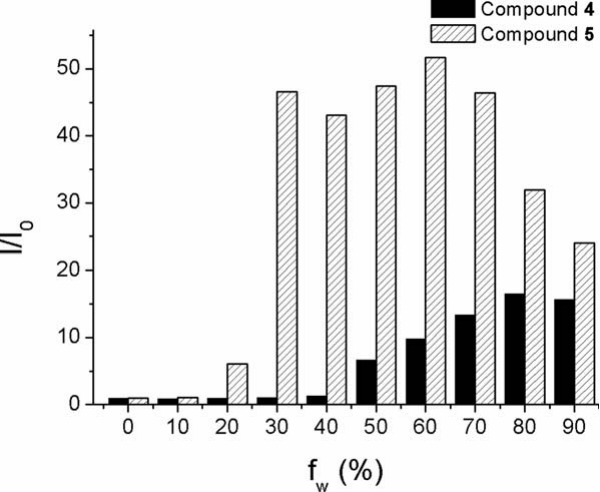
Intensity of the emission of compounds **4** and **5** in water:THF solutions relative to that in pure THF (I/I_0_) versus the water fraction (*f*_w_).

The AIE data for both compounds **4** and **5** are conventionally presented (Fig. 8) as a plot of the increase in the emission intensity of each solution relative to that of the solution in THF (I/I_0_) versus the water fraction of each solution.

#### Lasing and Piezofluorochromic Properties

Amplified spontaneous emission (ASE) experiments for compound **4** were performed on films spin‐cast from THF solutions of the material onto acetate sheet substrates covered with an epoxy (NOA65), and the films were left to dry at room temperature overnight. The conditions of the film deposition were varied in order to find those most suitable to make lasers. The spin‐coating was performed at: 3200 rpm (experiment 1) and 1000 rpm (experiment 2) from 30 mg/mL solutions, and 1000 rpm from a 50 mg/mL solution (experiment 3). For the first, second and third experiments, the ASE peaks were at 503, 505 and 509 nm, with ASE threshold values being 40, 140, and 540 µJ/cm^2^, respectively (Supporting Information Fig. S10). An increase in the threshold and the red‐shift of the ASE peak in experiments 1–3 are due to the different film thicknesses, which increased in the order: experiment 1 < experiment 2 < experiment 3.

**Figure 9:**
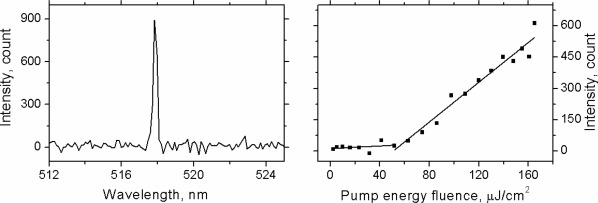
Spectrum (left) and power transfer function (right) of the laser.

A single transverse mode second‐order DFB laser was obtained by spin‐coating 30 mg/mL solutions of the material at 4000 rpm onto an epoxy grating (NOA65) with 330 nm periodicity (*Λ*). The thickness of the films under these spin‐coating conditions was 110 ± 10 nm (measured by AFM). The overall average threshold for this laser was 60 ± 6 µJ/cm^2^. This threshold value is the average of three measurements taken on the same laser, within an area of 3 × 3 mm. The results of one of the experiments are presented in Figure 9. The spectrum of the laser is shown at the pump energy fluence three times above threshold and presents a single peak at 517.9 nm with a FWHM of 0.26 nm determined by a Gaussian fit of the spectrum.

A series of studies were carried out to check the piezofluorochromic behavior of compound **4**. The effect of pressure on the ASE of films of compound **4** was first studied using a hydraulic press. Different pressures were applied to the films spin‐coated on NOA65 substrates and the ASE was measured before and after applying the pressure as shown in Supporting Information Figure S11. The pressure was applied on a 1 cm^2^ film of the material. Blue shifts in wavelength of 15.7, 19.1, 20.9, and 28.5 nm were obtained when applying, respectively, 200 MPa (2 tons/cm^2^), 250 MPa (2.5 tons/cm^2^), 300 MPa (3 tons/cm^2^), and 500 MPa (5 tons/cm^2^).

Most of the AIE‐active piezofluorochromic materials reported in the literature are compounds that exhibit a reasonably stable crystalline state, but the transition from this state to the amorphous one is possible under mechanical stimuli.^42^ Normally, the crystalline state provides a more twisted conformation of the AIE‐molecule compared to the amorphous one, and the transition under pressure or grinding is accompanied by a bathochromic shift. It is interesting to note that even for different oligomers from the same family the piezofluorochromic properties might be exhibited by one compound and can be completely lost by another. The series of hexagonal compounds **13** and **14** (Supporting Information Fig. S12) presents an example of this kind of behavior.^43^ While compound **13** in the ground powder form exhibited crystallization in the first heating cycle of DSC and a significantly red‐shifted fluorescence (*λ*_max_ = 497 nm) compared to the pristine crystalline form (*λ*_max_ = 467 nm), the oligomer with longer arms (**14**) was found to be less crystalline both in pristine and ground states and did not reveal any piezofluorochromic properties. Despite the absence of piezofluorochromic behavior, compound **14** exhibited more pronounced AIE‐activity and showed higher PLQY in the solid state compared to compound **13**. It proves that an increase in AIE‐activity might not be accompanied by improving the piezofluorochromic behavior.

**Figure 10:**
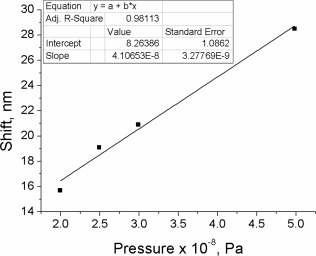
Blue shifts of the ASE peaks obtained after applying different pressures on films of compound **4** with a hydraulic press.

In contrast to all known examples of AIE‐active piezofluorochromic materials, none of the compounds **4–7** revealed any crystallinity. According to DSC all of them are amorphous solids, which is consistent with their star‐shaped architecture. The hypsochromic shift observed in the ASE experiment is likely to originate from the greater constraint of the molecules after applying pressure, which hindered the structural relaxation of the excited state, leading to an increase in the energy of emission. In this case, the change in the optical characteristics is not as pronounced as for the aforementioned AIE‐active materials that exhibit a morphological transition upon applying pressure. However, compound **4** does not require an exposure to a solvent vapor or thermal annealing in order to revert back into the initial state, and the effect of the pressure is completely reversible without any additional treatment. The sample that received a pressure of 200 MPa was left overnight and pumped the day after, showing a complete recovery of its ASE spectrum (Supporting Information Fig. S13a). Furthermore, the relationship between the spectral shift observed in the ASE and the value of pressure applied is linear and, therefore, compound **4** is a promising material for ASE‐based pressure sensing devices (Fig. 10).

In order to make sure that compound **4** was responsible for the spectral shift obtained after the application of pressure, and that this effect did not occur with other non‐AIE‐active gain materials, the same test was attempted with the tris(terfluorenyl)truxene (**T3**).^36^ A pressure of 200 MPa was applied on a film of **T3** spin‐coated on NOA65. Supporting Information Figure S13b shows the ASE spectrum of **T3** before and after the application of pressure. A blue shift of only 1 nm was found, probably due to small defects on the surface of the sample or variation of the thickness of the **T3** film. For the same pressure applied on compound **4**, a shift in wavelength of 15.7 nm was found. This result confirms that the shifts obtained for compound **4** were due to the arrangement of the molecules after applying a pressure and not to a film effect such as changes in thickness or in refractive index for example.

For more accurate measurements of the influence of the pressure on the emission of the film at lower pressure values, a torque press was used. Torque values of 10, 20, 30 and 40 ft·lbs were set on the wrench allowing deduction of the pressure applied on the sample. To calculate the pressure on the sample, the following formula (eq (1)) was used:

(1) $$P=\frac{T}{kdA}$$

with *T ‐* the torque (N·m), *k ‐* the torque coefficient (0.36 for steel), *d ‐* the diameter of the top of the screw (1.6 × 10^−2^ m) and *A ‐* the area of the sample (2.3 × 10^−2^ m by 4.2 × 10^−2^ m = 9.66 × 10^−4^ m^2^).

**Figure 11:**
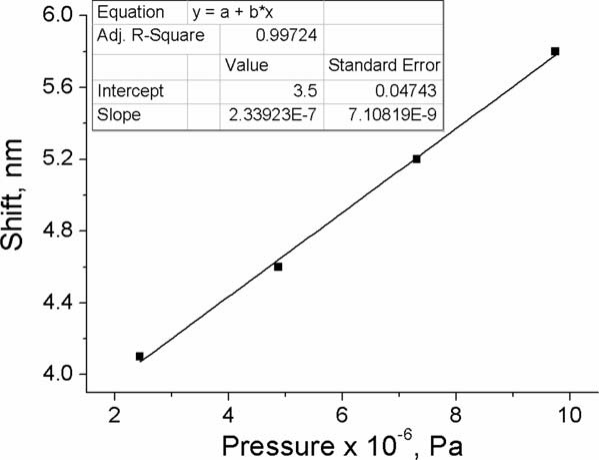
Blue shifts of the ASE peaks obtained after applying different pressures on compound **4** films with a torque press.

The estimated values of pressure were 2.44 MPa (244 N/cm^2^), 4.87 MPa (487 N/cm^2^), 7.31 MPa (731 N/cm^2^), and 9.75 MPa (975 N/cm^2^). These pressure values gave hypsochromic shifts in wavelength of 4.1, 4.6, 5.2, and 5.8 nm, respectively. The ASE spectra measured before and after applying the pressure are shown in Supporting Information Figure S14. The shifts are plotted as a function of the applied pressures in Figure 11. Within that range of pressure, the relationship is linear. The observed shift in wavelength was continuous and reproducible.

The ASE threshold was measured before and after the pressure was applied. The average threshold before applying the pressure corresponded to a pump energy fluence of 720 μJ/cm^2^. This value is higher than the value found previously using the same film deposition conditions and could be due to the reproducibility issues of the film morphology. After applying different pressures to the film, the threshold varied between 467 and 684 μJ/cm^2^. There was no trend following the threshold values but only random variations.

## CONCLUSIONS

In conclusion, a series of novel TPE‐cored star‐shaped oligofluorene materials (**4–7**) were synthesized. The materials are very poor emitters in solution but become efficient yellow‐green luminogens in the condensed state. They have a large absorption cross‐section and large Stokes shifts, which makes them promising candidates for down‐converter applications. Among the whole series the highest Stokes shift of 182 nm was observed for compound **4**.

Their AIE properties were investigated in water:THF mixtures in which compound **4** showed emission enhancement after the addition of 50% of water to the mixture, being maximized with 80% water content. The effect is observed at 20% water content for compound **5** and is maximized at 30% of water due to its lower solubility in polar solvents. The TPA containing oligomer **7** revealed the enhancement at 40%, reaching maximum emission intensity at 90% water content.

An all‐organic second‐order DFB laser was made using compound **4** as a gain material. It operated in a single transverse mode, with an average threshold energy fluence of 60 ± 6 μJ/cm^2^ and emission at 517.9 nm. The piezofluorochromic properties of **4** were also tested by studying the influence of pressure on the ASE of a thin film. A linear variation on a given range of the emission wavelength of **4** was found when applying different pressures, opening opportunities for development of lasing‐based pressure sensors.

## Supporting information

Supporting Information
