# Supplementary material for: Fluorene‐containing tetraphenylethylene molecules as lasing materials
Source: J Polym Sci A Polym Chem. 2016 Nov 21;55(4):734–46. doi: 10.1002/pola.28421 (PMC5516184; doi:10.1002/pola.28421)
Supplement: Supplementary file 1 — Supporting Information [file POLA-55-734-s001.docx]

Supporting Information

**C. Orofino,** ***^a^* C. Foucher,*^b^* F. Farrell,*^a^* N. J. Findlay,*^a^* B. Breig,*^a^* A. L. Kanibolotsky,*^ac^* B. Guilhabert,*^b^* F. Vilela,*^d^* N. Laurand,*^b^* M. D. Dawson*^c^* and P. J. Skabara**^a^***

*a* WestCHEM, Department of Pure and Applied Chemistry, University of Strathclyde, 295 Cathedral Street, Glasgow, G1 1XL, UK

*b* Institute of Photonics, Department of Physics, University of Strathclyde, Glasgow, UK

*c* Institute of Physical-Organic Chemistry and Coal Chemistry, 02160 Kyiv, Ukraine

*d* School of Engineering and Physical Sciences; Chemical Sciences, Heriot Watt University, Edinburgh, EH14 4AS, UK

Correspondence to: P. J. Skabara (E-mail: [*peter.skabara@strath.ac.uk*](mailto:peter.skabara@strath.ac.uk))

Figure S1 Thermal gravimetric analysis (TGA) of compounds **4-7**, with the decomposition temperatures (Td, 5% mass loss) marked.

Figure S2 Differential scanning calorimetry (DSC) of compounds **4-7**, with the glass transition temperatures T_g_ marked.

Figure S3 Cyclic voltammetry of compounds **4-7**, with the positions of peaks and onsets marked. Experimental conditions: scan rate – 0.1 V·s^-1^, solvent –acetonitrile : benzene (1 : 2) mixture, supporting electrolyte – 0.1 M tetrabutylammonium hexafluorophosphate ((TBA) PF_6_). A carbon disc (d=3 mm), Pt wire and Ag wire were used as working, counter and pseudo-reference electrodes, respectively. The electrochemical data are referenced against the Fc/Fc^+^ redox couple.

Figure S4 Cyclic voltammetry of compounds **6** and **7**. Scan rate – 0.1 V/s, solvent – CH_2_Cl_2_, supporting electrolyte – 0.1 M (TBA) PF_6_. Carbon disc (d=3 mm), Pt and Ag wire were used as working, counter and pseudo-reference electrodes respectively. The electrochemical data are referenced against the Fc/Fc+ redox couple.

Figure S5 The spectra (extinction coefficient (ε) *vs* wavelength) of compounds **4** and **5** in different solvents. The positions of the peaks and onsets are marked.

Figure S6 PL spectra of compounds **4** and **5** in dichloromethane and hexane at different concentrations. *λ*_ex_ (compound **4** in CH_2_Cl_2_) = 329 nm, *λ*_ex_ (compound **5** in CH_2_Cl_2_) = 372 nm, *λ*_ex_ = (compound **4** in hexane) = 327 nm, *λ*_ex_ (compound **5** in hexane) = 367 nm. The positions of the peaks are marked.

| 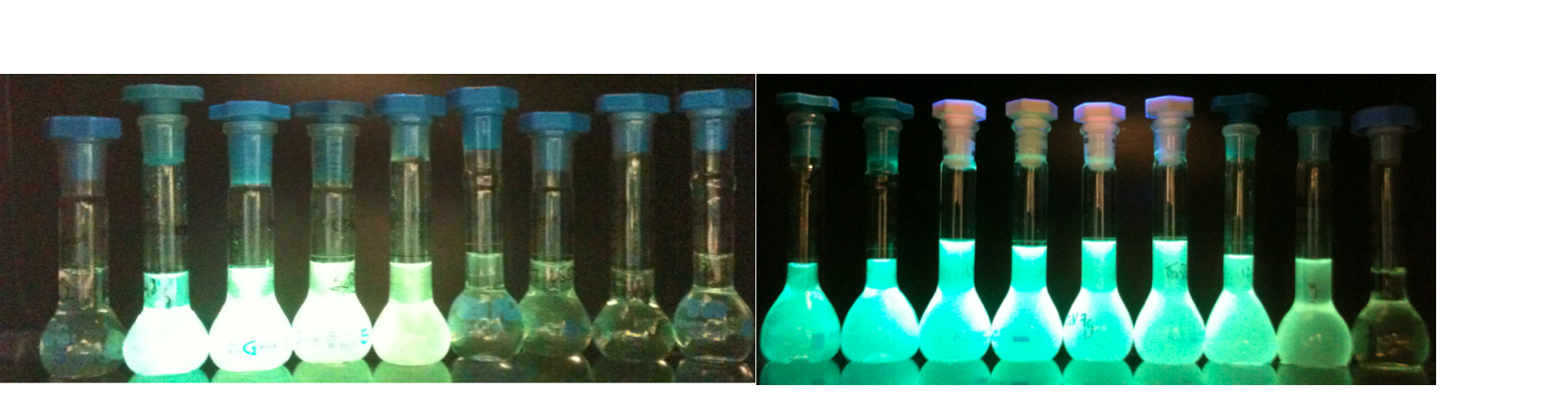 a  b |
| --- |
|  |

Figure S7 10 μM solutions of **4** (a) and **5** (b) in THF:H_2_O mixtures with increasing water fractions under UV (365 nm) illumination (from right to left 10 %, 20 %, 30 %, 40 %, 50 %, 60 %, 70 %, 80 % and 90 % water contents); c) absorption spectra of 10^-5^ M solutions of **4** in THF:H_2_O mixtures with increasing water fraction.

Figure S8 Absorption spectra of 10^-5^ M solutions of **5** in THF:H_2_O mixtures with increasing water fractions.

| *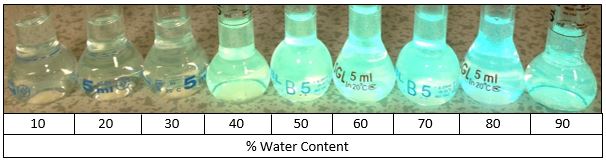* |
| --- |
| 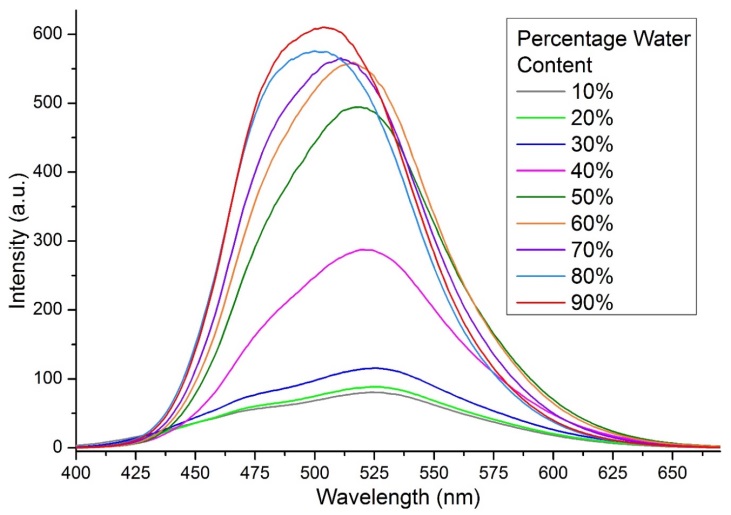 |

Figure S9 Top: images of 1 μM solutions of **7** in THF:H_2_O mixtures with increasing water fractions under UV (365 nm) illumination; bottom: emission spectra of these solutions, *λ*_ex_ = 369 nm.

Figure S10 Spectra and power transfer function for ASE of samples made with: a) a concentration of 30 mg/mL of compound **4** in THF spin-coated at 3200 rpm, b) a concentration of 30 mg/mL of compound **4** in THF spin-coated at 1000 rpm and c) a concentration of 50 mg/mL of compound **4** in THF spin-coated at 1000 rpm.

Figure S11 Piezofluorochromic studies on the ASE of compound **4** films using a hydraulic press, applying a pressure of a) 2 tons/cm^2^, b) 2.5 tons/cm^2^, c) 3 tons/cm^2^ and d) 5 tons/cm^2^.

Figure S12 Hexagonal star-shaped systems with a benzene core and TPE-units in the terminal positions.

|  |  |
| --- | --- |

Figure S13 a) ASE of compound **4** film showing the reversibility of the piezofluorochromic properties after only one night of the application of pressure (2 tons/cm^2^); b) ASE spectra of T3 before and after the application of pressure (2 tons/cm^2^) with a manual hydraulic press.

Figure S14 Piezofluorochromic studies on the ASE of compound **4** films using a torque press, applying a pressure of a) 2.44 MPa, b) 4.87 MPa, c) 7.31 MPa and d) 9.75 MPa.

Figure S15 ^1^H NMR spectrum of compound **4** in CD_2_Cl_2_.

Figure S16 ^13^C NMR spectrum of compound **4** in CD_2_Cl_2_.

Figure S17 ^1^H NMR spectrum of compound **5** in CDCl_3_.

Figure S18 ^13^C NMR spectrum of compound **5** in CDCl_3_.

Figure S19 ^1^H NMR spectrum of compound **6** in CDCl_3_.

Figure S20 ^13^C NMR spectrum of compound **6** in CDCl_3_.

Figure S21 ^1^H NMR spectrum of compound **7** in CDCl_3_.

Figure S22 ^13^C NMR spectrum of compound **7** in CDCl_3_.

Figure S23 ^1^H NMR spectrum of compound **12** in CDCl_3_.
